# Supplementary figures and images for: Opioid-related overdose and chronic use following an initial prescription of hydrocodone versus oxycodone
Source: PLoS One. 2022 Apr 5;17(4):e0266561. doi: 10.1371/journal.pone.0266561 (PMC8982846; doi:10.1371/journal.pone.0266561)

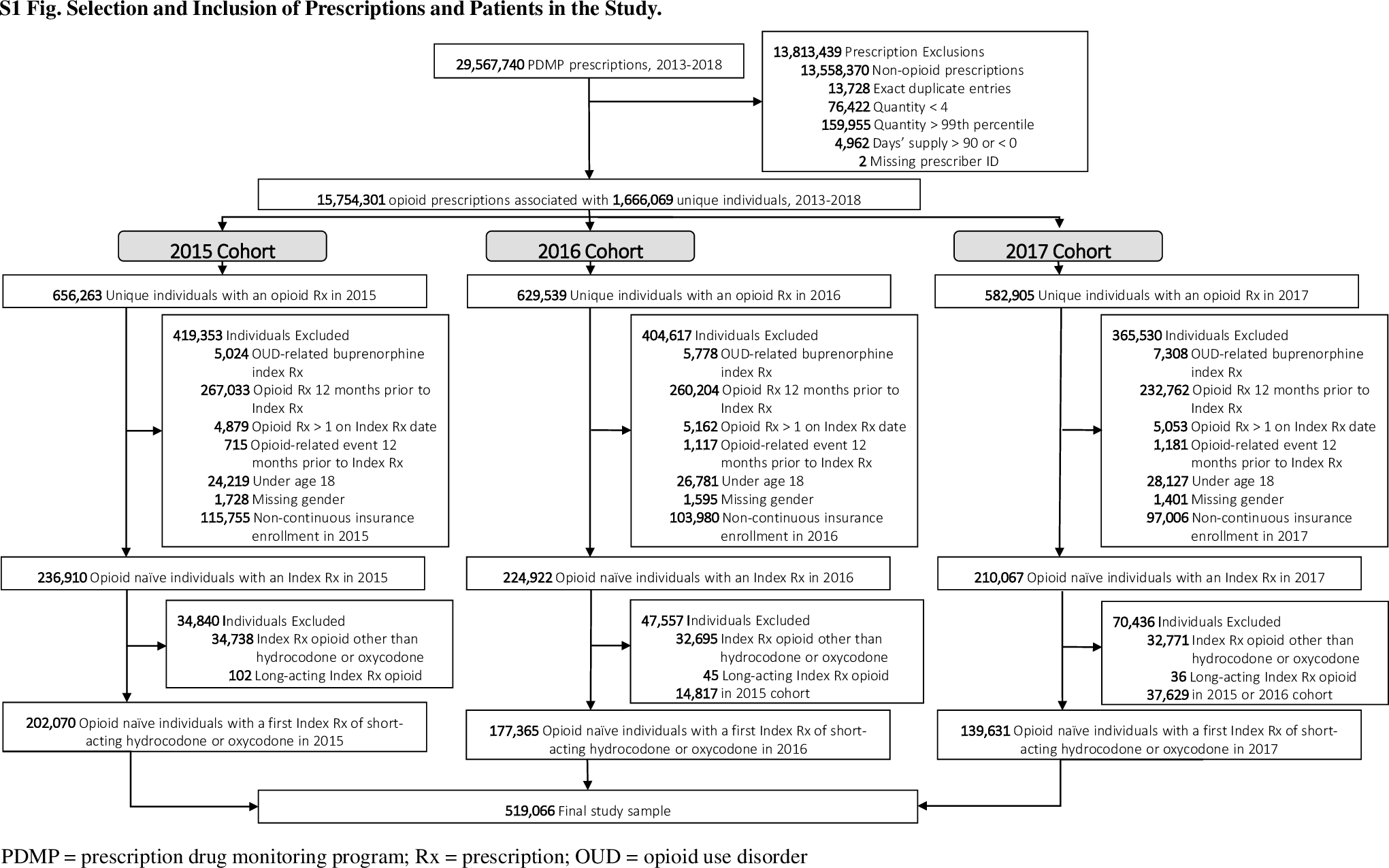

Supplement: S1 Fig — (TIF) [file pone.0266561.s001.tif]
