# Supplementary material for: Opioid-related overdose and chronic use following an initial prescription of hydrocodone versus oxycodone
Source: PLoS One. 2022 Apr 5;17(4):e0266561. doi: 10.1371/journal.pone.0266561 (PMC8982846; doi:10.1371/journal.pone.0266561)
Supplement: S1 Table — (DOCX) [file pone.0266561.s002.docx]

**S1 Table.** **ICD-9 or 10 diagnosis codes used to identify opioid-related overdose/poisoning for ED visits and inpatient hospitalization.**

|  | | |
| --- | --- | --- |
| ICD-9 | *Poisoning by:*  opium  heroin  methadone  other opiates and related narcotics  *Accidental poisoning by:*  heroin  methadone  other opiates and related narcotics | 96500  96501  96502  96509  E8500  E8501  E8502 |
| ICD-10 | *Poisoning by:*  opium  heroin  natural or semi-synthetic opioids  methadone  synthetic opioids, other than methadone  other and unspecified narcotics | T40.0  T40.1  T40.2  T40.3  T40.4  T40.6 |
