# Supplementary material for: Opioid-related overdose and chronic use following an initial prescription of hydrocodone versus oxycodone
Source: PLoS One. 2022 Apr 5;17(4):e0266561. doi: 10.1371/journal.pone.0266561 (PMC8982846; doi:10.1371/journal.pone.0266561)
Supplement: S2 Table — (DOCX) [file pone.0266561.s003.docx]

**S2 Table.** **Sensitivity Analysis: Patient and Index Prescription Characteristics Associated with First-year Chronic Use, Using an Alternative Chronic Use Definition.**

|  | **No Chronic Use (n = 502,769)** | **Chronic Use  (n = 16,297)** | **Chronic Use Row %  (3.14%)** | **Adjusted Odds Ratio (95% Confidence Interval)** | ***p* value** |
| --- | --- | --- | --- | --- | --- |
| **Index Prescription Drug** |  |  |  |  |  |
| **Hydrocodone SA** | 342,724 | 10,229 | 2.90 | ref | ref |
| **Oxycodone SA** | 160,045 | 6,068 | 3.65 | 0.91 (0.87-0.95) | <.0001 |
| **Age** |  |  |  |  |  |
| **18-24** | 65,476 | 634 | 0.96 | Ref | ref |
| **25-34** | 104,332 | 2,037 | 1.92 | 1.79 (1.64-1.96) | <.0001 |
| **35-44** | 88,011 | 2,563 | 2.83 | 2.63 (2.40-2.88) | <.0001 |
| **45-54** | 86,574 | 3,578 | 3.97 | 3.48 (3.18-3.80) | <.0001 |
| **55-64** | 86,533 | 3,917 | 4.33 | 3.67 (3.36-4.02) | <.0001 |
| **65-74** | 46,187 | 2,025 | 4.20 | 2.79 (2.49-3.12) | <.0001 |
| **75+** | 25,656 | 1,543 | 5.67 | 2.95 (2.62-3.32) | <.0001 |
| **Gender** |  |  |  |  |  |
| **Female** | 277,785 | 8,703 | 3.04 | 1.00 (0.97-1.035) | 0.99 |
| **Male** | 224,984 | 7,594 | 3.27 | ref | ref |
| **Race/Ethnicity** |  |  |  |  |  |
| **White** | 368,726 | 12,854 | 3.37 | ref | ref |
| **Black** | 15,782 | 601 | 3.67 | 1.17 (1.07-1.29) | 0.00 |
| **Hispanic** | 46,408 | 942 | 1.99 | 0.61 (0.56-0.65) | <.0001 |
| **Asian-Pacific Islander** | 12,375 | 148 | 1.18 | 0.38 (0.32-0.45) | <.0001 |
| **Other** | 7,570 | 338 | 4.27 | 1.08 (0.95-1.22) | 0.24 |
| **Unknown** | 51,908 | 1,414 | 2.65 | 0.91 (0.85-0.97) | 0.006 |
| **Insurance Plan in Index Year** |  |  |  |  |  |
| **Commercial** | 228,345 | 4,063 | 1.75 | Ref | ref |
| **Medicaid** | 189,492 | 7,480 | 3.80 | 2.79 (2.67-2.92) | <.0001 |
| **Medicare** | 64,348 | 2,683 | 4.00 | 1.633 (1.51-1.77) | <.0001 |
| **Dual** | 20,386 | 2,067 | 9.21 | 3.66 (3.40-3.93) | <.0001 |
| **Unknown** | 198 | 4 | 1.98 | 1.24 (0.43-3.52) | 0.69 |
| **Urbanization in Index Year** |  |  |  |  |  |
| **Large central metro** | 91,017 | 2,672 | 2.85 | 0.85 (0.74-0.99) | 0.04 |
| **Large fringe metro** | 111,484 | 3,142 | 2.74 | 0.91 (0.78-1.05) | 0.19 |
| **Medium metro** | 95,518 | 2,894 | 2.94 | 0.90 (0.77-1.05) | 0.17 |
| **Small metro** | 71,255 | 2,364 | 3.21 | 0.89 (0.77-1.04) | 0.14 |
| **Micropolitan** | 46,746 | 1,872 | 3.85 | 0.97 (0.83-1.13) | 0.70 |
| **Noncore** | 7,083 | 296 | 4.01 | Ref | ref |
| **Unknown** | 79,666 | 3,057 | 3.70 | 0.99 (0.85-1.14) | 0.85 |
| **Year of Index Prescription** |  |  |  |  |  |
| **2015** | 194,635 | 7,435 | 3.68 | ref | ref |
| **2016** | 172,443 | 4,922 | 2.78 | 0.83 (0.80-0.86) | <.0001 |
| **2017** | 135,691 | 3,940 | 2.82 | 0.87 (0.83-0.91) | <.0001 |
| **Index Prescription MME** |  |  |  |  |  |
| **MME <= 75** | 149,413 | 2,936 | 1.93 | ref | ref |
| **MME 76-100** | 106,999 | 1,986 | 1.82 | 1.01 (0.95-1.08) | 0.70 |
| **MME 101-200** | 124,153 | 3,829 | 2.99 | 1.47 (1.38-1.56) | <.0001 |
| **MME 201-300** | 81,969 | 3,276 | 3.84 | 1.89 (1.76-2.03) | <.0001 |
| **MME >300** | 40,235 | 4,270 | 9.59 | 3.98 (3.66-4.32) | <.0001 |
| **Index Prescription Days' Supply** |  |  |  |  |  |
| **<= 3 Days** | 286,762 | 5,269 | 1.80 | ref | ref |
| **4 - 6 Days** | 147,475 | 3,557 | 2.36 | 1.06 (1.01-1.11) | 0.033 |
| **7+ Days** | 68,532 | 7,471 | 9.83 | 2.60 (2.46-2.75) | <.0001 |

SA, short-acting; MME, morphine milligram equivalents
