# Supplementary material for: Opioid-related overdose and chronic use following an initial prescription of hydrocodone versus oxycodone
Source: PLoS One. 2022 Apr 5;17(4):e0266561. doi: 10.1371/journal.pone.0266561 (PMC8982846; doi:10.1371/journal.pone.0266561)
Supplement: S3 Table — (DOCX) [file pone.0266561.s004.docx]

**S3 Table.** **Multilevel Cox Regression Sub-analysis: Patient and Index Prescription Characteristics Associated with Opioid Overdose.**

|  | **No Overdose  (n = 101,559)** | **Overdose (n = 287)** | **Overdose Row % (0.28%)** | **Adjusted Hazard Ratio (95% Confidence Interval)** | ***p* value** |
| --- | --- | --- | --- | --- | --- |
| **Index Prescription Drug** |  |  |  |  |  |
| **Hydrocodone SA** | 66,747 | 163 | 0.24 | ref | ref |
| **Oxycodone SA** | 34,812 | 124 | 0.35 | 1.75 (1.29-2.37) | 0.0003 |
| **Age** |  |  |  |  |  |
| **18-24** | 18,725 | 70 | 0.37 | Ref | ref |
| **25-34** | 22,261 | 76 | 0.34 | 0.75 (0.54-1.04) | 0.087 |
| **35-44** | 16,863 | 50 | 0.30 | 0.69 (0.48-0.99) | 0.042 |
| **45-54** | 16,178 | 35 | 0.22 | 0.52 (0.34-0.78) | 0.002 |
| **55-64** | 15,698 | 25 | 0.16 | 0.47 (0.29-0.75) | 0.001 |
| **65-74** | 7,787 | 19 | 0.24 | 0.76 (0.35-1.65) | 0.489 |
| **75+** | 4,047 | 12 | 0.30 | 0.99 (0.41-2.40) | 0.989 |
| **Gender** |  |  |  |  |  |
| **Female** | 55,054 | 112 | 0.20 | 0.99 (0.41-2.40) | <.0001 |
| **Male** | 46,505 | 175 | 0.37 | ref | ref |
| **Race/Ethnicity** |  |  |  |  |  |
| **White** | 73,946 | 238 | 0.32 | ref | ref |
| **Black** | 3,664 | 10 | 0.27 | 0.58 (0.31-1.12) | 0.10 |
| **Hispanic** | 10,371 | 19 | 0.18 | 0.46 (0.29-0.74) | 0.001 |
| **Asian-Pacific Islander** | 2,758 | 3 | 0.11 | 0.31 (0.10-0.97) | 0.04 |
| **Other** | 1,514 | 8 | 0.53 | 1.10 (0.54-2.22) | 0.80 |
| **Unknown** | 9,306 | 9 | 0.10 | 0.44 (0.22-0.89) | 0.02 |
| **Insurance Plan in Index Year** |  |  |  |  |  |
| **Commercial** | 40,339 | 38 | 0.09 | Ref | ref |
| **Medicaid** | 46,431 | 207 | 0.44 | 4.34 (3.04-6.21) | <.0001 |
| **Medicare** | 10,463 | 23 | 0.22 | 1.88 (0.83-4.25) | 0.13 |
| **Dual** | 4,281 | 19 | 0.44 | 4.13 (2.24-7.63) | <.0001 |
| **Unknown** | 45 | 0 | 0.00 | 0.001 (0-3.31E+132) | 0.96 |
| **Urbanization in Index Year** |  |  |  |  |  |
| **Large central metro** | 19,213 | 66 | 0.34 | ref | ref |
| **Large fringe metro** | 21,820 | 52 | 0.24 | 0.79 (0.55-1.15) | 0.22 |
| **Medium metro** | 24,345 | 68 | 0.28 | 0.85 (0.60-1.21) | 0.37 |
| **Small metro** | 14,552 | 38 | 0.26 | 0.77 (0.51-1.15) | 0.20 |
| **Micropolitan** | 5,736 | 18 | 0.31 | 0.77 (0.45-1.31) | 0.33 |
| **Noncore** | 957 | 0 | 0.00 | 0.00 (0.00-0.00) | - |
| **Unknown** | 14,936 | 45 | 0.30 | 1.00 (0.67-1.48) | 0.98 |
| **Year of Index Prescription** |  |  |  |  |  |
| **2015** | 39,874 | 139 | 0.35 | ref | ref |
| **2016** | 35,894 | 98 | 0.27 | 1.14 (0.87-1.50) | 0.34 |
| **2017** | 25,791 | 50 | 0.19 | 1.32 (0.93-1.87) | 0.12 |
| **Index Prescription MME** |  |  |  |  |  |
| **MME <= 75** | 30,152 | 95 | 0.31 | ref | ref |
| **MME 76-100** | 23,350 | 58 | 0.25 | 0.84 (0.61-1.18) | 0.31 |
| **MME 101-200** | 29,342 | 84 | 0.29 | 0.76 (0.53-1.08) | 0.12 |
| **MME 201-300** | 13,446 | 30 | 0.22 | 0.64 (0.39-1.05) | 0.08 |
| **MME >300** | 5,269 | 20 | 0.38 | 0.86 (0.46-1.61) | 0.64 |
| **Index Prescription Days Supply** |  |  |  |  |  |
| **<= 3 Days** | 66,345 | 195 | 0.29 | ref | ref |
| **4 - 6 Days** | 29,020 | 66 | 0.23 | 0.81 (0.60-1.10) | 0.18 |
| **7+ Days** | 6,194 | 26 | 0.42 | 1.40 (0.87-2.26) | 0.16 |

SA, short-acting; MME, morphine milligram equivalents
