# Supplementary material for: Opioid-related overdose and chronic use following an initial prescription of hydrocodone versus oxycodone
Source: PLoS One. 2022 Apr 5;17(4):e0266561. doi: 10.1371/journal.pone.0266561 (PMC8982846; doi:10.1371/journal.pone.0266561)
Supplement: S4 Table — (DOCX) [file pone.0266561.s005.docx]

**S4 Table.** **Combination Drug Sub-analysis: Patient and Index Prescription Characteristics Associated with First-year Chronic Use.**

|  | **No Chronic Use (n = 466,129)** | **Chronic Use  (n = 12,004)** | **Chronic Use Row %  (2.5%)** | **Adjusted Odds Ratio (95% Confidence Interval)** | ***p* value** |
| --- | --- | --- | --- | --- | --- |
| **Index Prescription Drug** |  |  |  |  |  |
| **Hydrocodone-Acetaminophen SA** | 312,663 | 7,279 | 2.28 | ref | ref |
| **Oxycodone-Acetaminophen SA** | 73,988 | 1,822 | 2.40 | 0.88 (0.83-0.94) | 0.000 |
| **Oxycodone Monotherapy SA** | 79,478 | 2,903 | 3.52 | 1.06 (1.00-1.13) | 0.055 |
| **Age** |  |  |  |  |  |
| **18-24** | 60,982 | 501 | 0.81 | ref | ref |
| **25-34** | 98,086 | 1,527 | 1.53 | 1.74 (1.57-1.93) | <.0001 |
| **35-44** | 81,865 | 1,903 | 2.27 | 2.64 (2.39-2.92) | <.0001 |
| **45-54** | 79,591 | 2,544 | 3.10 | 3.47 (3.14-3.83) | <.0001 |
| **55-64** | 78,924 | 2,856 | 3.49 | 3.84 (3.47-4.25) | <.0001 |
| **65-74** | 42,459 | 1,494 | 3.40 | 3.10 (2.73-3.52) | <.0001 |
| **75+** | 24,222 | 1,179 | 4.64 | 3.34 (2.93-3.81) | <.0001 |
| **Gender** |  |  |  |  |  |
| **Female** | 260,381 | 6,454 | 2.42 | 0.96 (0.92-1.00) | 0.026 |
| **Male** | 205,748 | 5,550 | 2.63 | ref | ref |
| **Race/Ethnicity** |  |  |  |  |  |
| **White** | 341,085 | 9,467 | 2.70 | ref | ref |
| **Black** | 15,122 | 462 | 2.96 | 1.17 (1.06-1.30) | 0.003 |
| **Hispanic** | 43,843 | 701 | 1.57 | 0.59 (0.54-0.64) | <.0001 |
| **Asian-Pacific Islander** | 11,791 | 115 | 0.97 | 0.36 (0.30-0.44) | <.0001 |
| **Other** | 7,104 | 245 | 3.33 | 1.11 (0.97-1.28) | 0.138 |
| **Unknown** | 47,184 | 1,014 | 2.10 | 0.89 (0.83-0.97) | 0.005 |
| **Insurance Plan in Index Year** |  |  |  |  |  |
| **Commercial** | 209,649 | 3,012 | 1.42 | Ref | ref |
| **Medicaid** | 177,667 | 5,514 | 3.01 | 2.81 (2.67-2.95) | <.0001 |
| **Medicare** | 59,635 | 1,969 | 3.20 | 1.51 (1.38-1.65) | <.0001 |
| **Dual** | 18,999 | 1,505 | 7.34 | 3.63 (3.35-3.94) | <.0001 |
| **Unknown** | 179 | 4 | 2.19 | 1.61 (0.57-4.56) | 0.373 |
| **Urbanization in Index Year** |  |  |  |  |  |
| **Large central metro** | 87,065 | 2,102 | 2.36 | 0.86 (0.72-1.02) | 0.073 |
| **Large fringe metro** | 105,984 | 2,464 | 2.27 | 0.91 (0.77-1.08) | 0.293 |
| **Medium metro** | 89,023 | 2,168 | 2.38 | 0.90 (0.76-1.07) | 0.248 |
| **Small metro** | 64,220 | 1,634 | 2.48 | 0.89 (0.75-1.05) | 0.172 |
| **Micropolitan** | 40,262 | 1,204 | 2.90 | 0.97 (0.81-1.15) | 0.694 |
| **Noncore** | 6,453 | 200 | 3.01 | ref | ref |
| **Unknown** | 73,122 | 2,232 | 2.96 | 1.02 (0.86-1.20) | 0.852 |
| **Year of Index Prescription** |  |  |  |  |  |
| **2015** | 180,101 | 5,376 | 2.90 | ref | ref |
| **2016** | 159,868 | 3,692 | 2.26 | 0.84 (0.81-0.88) | <.0001 |
| **2017** | 126,160 | 2,936 | 2.27 | 0.86 (0.82-0.91) | <.0001 |
| **Index Prescription MME** |  |  |  |  |  |
| **MME <= 75** | 147,691 | 2,548 | 1.70 | ref | ref |
| **MME 76-100** | 104,782 | 1,732 | 1.63 | 1.01 (0.95-1.09) | 0.709 |
| **MME 101-200** | 112,290 | 3,187 | 2.76 | 1.47 (1.38-1.58) | <.0001 |
| **MME 201-300** | 71,258 | 2,444 | 3.32 | 1.76 (1.62-1.92) | <.0001 |
| **MME >300** | 30,108 | 2,093 | 6.50 | 2.93 (2.65-3.23) | <.0001 |
| **Index Prescription Days' Supply** |  |  |  |  |  |
| **<= 3 Days** | 272,984 | 4,471 | 1.61 | ref | ref |
| **4 - 6 Days** | 133,749 | 2,922 | 2.14 | 1.11 (1.05-1.18) | 0.0002 |
| **7+ Days** | 59,396 | 4,611 | 7.20 | 2.46 (2.31-2.62) | <.0001 |

SA, short-acting; MME, morphine milligram equivalents
