# Supplementary material for: Opioid-related overdose and chronic use following an initial prescription of hydrocodone versus oxycodone
Source: PLoS One. 2022 Apr 5;17(4):e0266561. doi: 10.1371/journal.pone.0266561 (PMC8982846; doi:10.1371/journal.pone.0266561)
Supplement: S5 Table — (DOCX) [file pone.0266561.s006.docx]

**S5 Table**. **Combination Drug Sub-analysis: Patient and Index Prescription Characteristics Associated with Opioid Overdose.**

|  | **No Overdose  (n = 476,787)** | **Overdose (n = 1,346)** | **Overdose Row % (0.28%)** | **Adjusted Hazard Ratio (95% Confidence Interval)** | ***p* value** |
| --- | --- | --- | --- | --- | --- |
| **Index Prescription Drug** |  |  |  |  |  |
| **Hydrocodone-Acetaminophen SA** | 319,164 | 778 | 0.24 | ref | ref |
| **Oxycodone-Acetaminophen SA** | 75,605 | 205 | 0.27 | 1.26 (1.06 - 1.50) | 0.0088 |
| **Oxycodone Monotherapy SA** | 82,018 | 363 | 0.44 | 2.18 (1.86-2.57) | <.0001 |
| **Age** |  |  |  |  |  |
| **18-24** | 61,248 | 235 | 0.38 | Ref | ref |
| **25-34** | 99,333 | 280 | 0.28 | 0.70 (0.59-0.83) | <.0001 |
| **35-44** | 83,573 | 195 | 0.23 | 0.64 (0.53-0.78) | <.0001 |
| **45-54** | 81,921 | 214 | 0.26 | 0.73 (0.60-0.88) | 0.001 |
| **55-64** | 81,592 | 188 | 0.23 | 0.74 (0.60-0.90) | 0.003 |
| **65-74** | 43,845 | 108 | 0.25 | 0.73 (0.53-1.01) | 0.054 |
| **75+** | 25,275 | 126 | 0.50 | 1.49 (1.09-2.04) | 0.014 |
| **Gender** |  |  |  |  |  |
| **F** | 266,175 | 660 | 0.25 | 0.68 (0.61-0.76) | <.0001 |
| **M** | 210,612 | 686 | 0.32 | ref | ref |
| **Race/Ethnicity** |  |  |  |  |  |
| **White** | 349,481 | 1,071 | 0.31 | ref | ref |
| **Black** | 15,521 | 63 | 0.40 | 0.91 (0.70-1.18) | 0.47 |
| **Hispanic** | 44,461 | 83 | 0.19 | 0.48 (0.38-0.60) | <.0001 |
| **Asian Pacific Islander** | 11,895 | 11 | 0.09 | 0.25 (0.14-0.45) | <.0001 |
| **Other** | 7,312 | 37 | 0.50 | 1.11 (0.80-1.55) | 0.53 |
| **Unknown** | 48,117 | 81 | 0.17 | 0.93 (0.72-1.18) | 0.54 |
| **Insurance Plan in Index Year** |  |  |  |  |  |
| **Commercial** | 212,466 | 195 | 0.09 | ref | ref |
| **Medicaid** | 182,337 | 844 | 0.46 | 5.18 (4.40-6.09) | <.0001 |
| **Medicare** | 61,442 | 162 | 0.26 | 2.02 (1.48-2.75) | <.0001 |
| **Dual** | 20,360 | 144 | 0.70 | 6.35 (4.93-8.18) | <.0001 |
| **Unknown** | 182 | 1 | 0.55 | 5.93 (0.83-42.34) | 0.08 |
| **Urbanization in Index Year** |  |  |  |  |  |
| **Large central metro** | 88,843 | 324 | 0.36 | ref | ref |
| **Large fringe metro** | 108,199 | 249 | 0.23 | 0.75 (0.64-0.89) | 0.00 |
| **Medium metro** | 90,927 | 264 | 0.29 | 0.86 (0.72-1.01) | 0.00 |
| **Small metro** | 65,678 | 176 | 0.27 | 0.74 (0.61-0.90) | 0.00 |
| **Micropolitan** | 41,361 | 105 | 0.25 | 0.65 (0.52-0.82) | 0.00 |
| **Noncore** | 6,641 | 12 | 0.18 | 0.49 (0.27-0.87) | 0.02 |
| **Unknown** | 75,138 | 216 | 0.29 | 0.80 (0.66-0.96) | 0.02 |
| **Year of Index Prescription** |  |  |  |  |  |
| **2015** | 184,836 | 641 | 0.35 | Ref | ref |
| **2016** | 163,116 | 444 | 0.27 | 1.06 (0.94-1.21) | 0.33 |
| **2017** | 128,835 | 261 | 0.20 | 1.22 (1.04-1.42) | 0.01 |
| **Index Prescription MME** |  |  |  |  |  |
| **MME <= 75** | 149,806 | 433 | 0.29 | Ref | ref |
| **MME 76-100** | 106,270 | 244 | 0.23 | 0.91 (0.78-1.07) | 0.26 |
| **MME 101-200** | 115,118 | 359 | 0.31 | 0.96 (0.82-1.14) | 0.66 |
| **MME 201-300** | 73,506 | 196 | 0.27 | 0.76 (0.61-0.94) | 0.01 |
| **MME >300** | 32,087 | 114 | 0.35 | 0.75 (0.57-0.99) | 0.05 |
| **Index Prescription Days Supply** |  |  |  |  |  |
| **<= 3 Days** | 276,659 | 796 | 0.29 | ref | ref |
| **4 - 6 Days** | 136,361 | 310 | 0.23 | 0.82 (0.71-0.95) | 0.01 |
| **7+ Days** | 63,767 | 240 | 0.37 | 1.33 (1.11-1.58) | 0.00 |

SA, short-acting; MME, morphine milligram equivalents
